# Fabrication of High-Performance Polyisocyanurate Aerogels through Cocyclotrimerization of 4,4′-Methylene Diphenyl Diisocyanate and Its Mono-Urethane Derivatives

**DOI:** 10.1021/acsami.4c07480

**Published:** 2024-06-26

**Authors:** Changlin Wang, Yunfei Guo, Tankut Türel, Željko Tomović

**Affiliations:** Polymer Performance Materials Group, Department of Chemical Engineering and Chemistry, Eindhoven University of Technology, 5600 MB Eindhoven, The Netherlands

**Keywords:** aerogel, polyisocyanurate, urethane, superinsulation, thermal stability

## Abstract

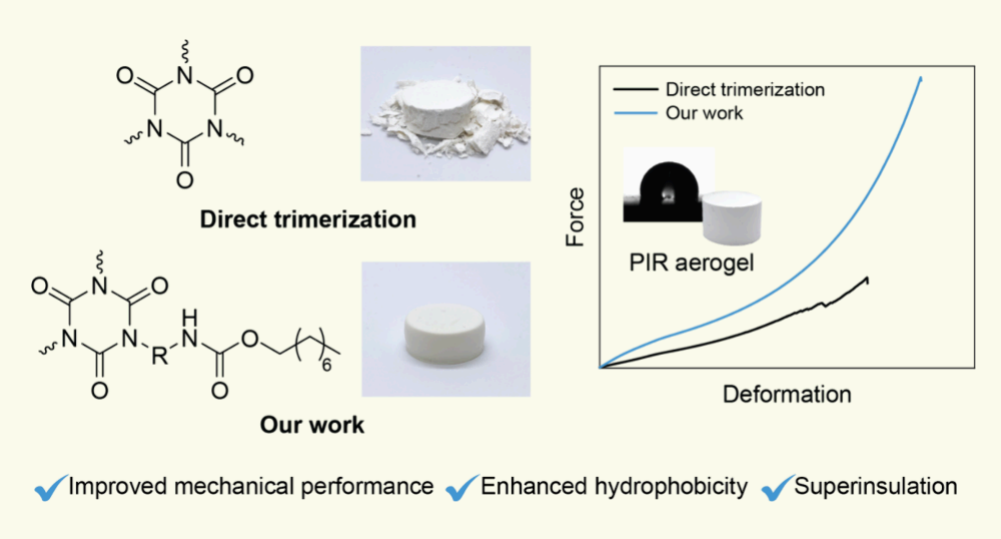

Aromatic polyisocyanurate (PIR) aerogels are recognized as advanced porous materials and extensively studied due to their lightweight nature, high porosity, and specific surface area, which attribute to their outstanding thermal insulation properties. The inherent thermal stability of the PIR moieties, combined with great insulating performance, renders PIR aerogels highly suitable for building insulation applications. Nevertheless, materials containing isocyanurate obtained through direct trimerization of aromatic isocyanates exhibit brittleness, resulting in inferior mechanical performance. In order to enhance the processability of the PIR aerogels, we propose a cocyclotrimerization approach involving mixtures of mono- and difunctional aromatic isocyanates. This approach is designed to develop a PIR network with decreased cross-linking density and brittleness. Herein, we developed an array of PIR aerogels from different alkyl chain-modified isocyanate mixtures. The resulting PIR aerogels exhibited high porosity (>89%), a large surface area (∼300 m^2^/g), superinsulating performance with ultralow thermal conductivity (∼16.8 mW m^–1^ K^–1^), notable thermal stability (*T*_d5%_ ∼ 250 °C), improved mechanical performance, and intrinsic hydrophobicity without the need for postmodification. These high-performance organic aerogels hold significant promise for applications requiring superinsulating materials.

## 1 Introduction

With the growing dependence of our society on energy, the reduction of energy consumption, energy costs, and associated environmental issues have gained extensive attention. It is noteworthy that around 40% of global energy usage is attributed to buildings, highlighting the importance of optimizing building energy consumption.^1,2^ The energy efficiency of the buildings can be enhanced by minimizing heat transfer to the external environment, a goal often achieved through enhancing the thermal insulation of the buildings. An improved insulation is attainable through reduction of the thermal conductivity of the building materials. For instance, aerogels are excellent candidates as thermal insulating materials, offering thermal conductivities below 0.020 W m^–1^ K^–1^, significantly lower than those of other commercially available materials such as wood fibers, mineral wools, and polymer foams.^3−5^ Aerogels possess nanoscopic pores with diameters shorter than the mean free path length of air, a characteristic that limits the mobility of gas molecules and hinders their collision (known as the Knudsen effect).^6,7^ Subsequently, the thermal conductivity of aerogels through the gas phase is strongly reduced, leading to superior thermal insulation relative to conventional polymeric foams.^3,5^

Organic aerogels represent the most versatile type of aerogels, featuring various polymer networks such as resorcinol-formaldehyde,^8,9^ polyurethane,^10−12^ polyurea,^13−16^ and polyimide.^17,18^ These aerogels typically demonstrate stronger mechanical properties compared to commercial silica aerogels, owing to the presence of covalent C–C bonds within their polymer networks.^19,20^ Nevertheless, organic aerogels commonly exhibit low thermal stability and poor flame resistance,^8,13^ making them less than ideal for building insulation applications. To address this limitation, thermally stable chemical moieties (e.g., imides,^21,22^ phosphazene,^23^ and isocyanurates^14,24−27^) have been incorporated into organic aerogels to improve their thermal properties.

Aromatic polyisocyanurate (PIR) stands out as one of the most thermally stable chemical moieties employed in polymer materials, characterized by its high thermal decomposition temperature (*T*_d5%_ above 270 °C).^28,29^ These highly cross-linked materials are typically obtained from trimerization of aromatic isocyanates.^25,26,29,30^ However, the inherent stiffness of the PIR structure often results in incomplete reactions as well as brittle materials. To counter this issue, researchers have explored cocyclotrimerization of mono- and difunctional isocyanate mixtures, resulting in a PIR-rich network with reduced cross-link density and brittleness.^31,32^ Materials derived from this mixture exhibited notable flexibility and possessed a high decomposition temperature (*T*_d5%_ > 400 °C). In another work, this approach was adopted to fabricate flexible PIR materials using mono- and difunctional isocyanate mixtures prepared from 2-ethyl-1-hexanol and 4,4′-methylene diphenyl diisocyanate (MDI).^33,34^ To take the development one step further, in this work we present a synthetic protocol to fabricate superinsulating, thermally stable, hydrophobic PIR aerogels with excellent mechanical performance. Initially, mixtures of mono- and difunctional isocyanates were synthesized through the reaction between MDI and primary monofunctional alcohols such as 2-ethyl-1-hexanol (C6,2) or 1-octanol (C8) in varying molar ratios. Subsequently, these mixtures were cocyclotrimerized to form stable organogels using potassium 2-ethylhexanoate (KEH) as a catalyst, followed by supercritical drying to obtain aerogels. The resulting PIR aerogels revealed low density (<0.16 g cm^–3^), high porosity (∼89%), high surface area (>300 m^2^ g^–1^), and ultralow thermal conductivity (∼16.8 mW m^–1^ K^–1^). Owing to their high PIR contents, these aerogels showed outstanding thermal stability, with *T*_d5%_ values exceeding 240 °C. By incorporating nonpolar alkyl chains during synthesis, our PIR aerogels demonstrated intrinsic hydrophobicity without the need for postmodification and excellent mechanical performance without exhibiting brittleness.

## 2 Experimental Section

### 2.1 Materials

2-Ethyl-1-hexanol (≥99.6%), *p*-tolyl isocyanate (99%), *n*-butanol (anhydrous), 1-octanol (anhydrous), and 3-pentanone were purchased from Merck Science B.V. Acetone-*d*_*6*_ (99.9% D) was obtained from Cambridge Isotope Laboratories. Silica gel, for chromatography, 0.030–0.200 mm, was purchased from Thermo Fischer Scientific Inc. 4,4′-Diamino-3,3′,5,5′-tetraethyldiphenylmethane (MDEA) and potassium 2-ethylhexanoate (KEH) were procured from TCI Europe B.V. Methylethylketone (MEK), heptane, and ethyl acetate were from Biosolve B.V. 4,4′-methylene diphenyl diisocyanate (MDI), oligomeric methylene diphenyl diisocyanate (Lupranat M200), and 85% potassium ethyl hexanoate dissolved in diethylene glycol (Dabco K-15) were provided by BASF Polyurethanes GmbH. Liquid CO_2_ (grade 2.7), N_2_ (grade 5.0), and He (grade 4.6) were purchased from Linde gas B.V.

### 2.2 Synthesis of Mono-/Difunctional Aromatic Isocyanate Mixtures

To prepare the aromatic isocyanate mixtures, MDI was reacted with either 2-ethyl-1-hexanol or 1-octanol in the molar ratios of 1 to 0.1 or 1 to 0.25. Detailed information on all synthesized samples is provided in the Supporting Information. Synthesis of isocyanate mixtures—MDI_C8_0.25: A mixture of MDI and 1-octanol in a molar ratio of 1:0.25 (MDI_C8_0.25) was employed. MDI (87.6 g, 0.35 mol) was added in a dry three-neck flask equipped with a dropping funnel and stirred under an Ar atmosphere at 50 °C. Subsequently, 1-octanol (11.4 g, 87.5 mmol) was slowly added dropwise into the flask using a dropping funnel, ensuring that the internal temperature remained below 55 °C. The reaction was stopped immediately after the addition was complete. The resulting mixture was collected and stored at −20 °C.

### 2.3 Synthesis of 2-Ethylhexyl-*p*-tolylcarbamate

*p*-Tolyl isocyanate (1.6 g, 12.18 mmol) and 2-ethyl-1-hexanol (1.8 g, 13.39 mmol) were dissolved in toluene (5 mL) in a three-neck flask equipped with a condenser. The mixture was stirred and heated to 50 °C under Ar until the disappearance of the NCO stretching band at 2270 cm^–1^, as observed through FTIR. Upon evaporation of the solvent, the final compound was obtained by column chromatography using silica gel as the stationary phase and heptane/ethyl acetate (9/1 v/v) as the eluent, resulting in the formation of a transparent liquid with a yield of 40%. ^1^H NMR (400 MHz, 25 °C, acetone-*d*_*6*_): δ= 8.49 (s, 1H), 7.44 (d, *J* = 8.2 Hz, 2H), 7.09 (d, 2H), 4.21–3.89 (m, 2H), 2.26 (s, 3H), 1.59 (hept, 1H), 1.51–1.17 (m, 8H), 1.00–0.81 (m, 6H) ppm; ^13^C NMR (400 MHz, 25 °C, acetone-*d*_*6*_): δ = 153.8, 136.9, 131.6, 129.1, 118.2, 66.4, 39.1, 30.2, 23.5, 22.8, 19.9, 13.5, 10.5 ppm (Figure S1).

### 2.4 Study of Trimerization Mechanism of *p*-Tolyl Isocyanate Using Potassium 2-Ethylhexoanate as Trimerization Catalyst

*p*-Tolyl isocyanate (32.7 mg, 0.13 mmol) and 2-ethylhexyl-*p*-tolylcarbamate (16.6 mg, 0.13 mol) were dissolved in 1.0 mL of acetone-*d*_6._ The reaction was initiated by introducing potassium 2-ethylhexaonate (0.47 mg, 0.0026 mmol) in 0.3 mL of acetone-*d*_*6*_ solution. Subsequent monitoring was performed by using ^1^H NMR spectroscopy at room temperature.

### 2.5 PIR Aerogel Preparation

The PIR organogel (**PIR-B2**) was prepared by mixing components A and B. Component A consisted of 4.99 g mono-/difunctional isocyanate mixtures (MDI-C8_0.25) dissolved in 13.5 g 3-pentanone, while component B contained 0.012 g of KEH dissolved in 13.5 g 3-pentanone. Both components were prepared in a polypropylene (PP) vial by dissolving monomers at room temperature. The gelation was initiated by mixing the two components into one vial, which was then shaken until a homogeneous solution was obtained. The solution was poured into PTFE mold with a diameter of 65 mm and then placed at room temperature for 8 h until gelation. Following gelation, the organogel was sealed and allowed to age for 24 h under ambient conditions. Subsequently, the organogel was then transferred into an autoclave, submerged in 3-pentanone, and sealed in a supercritical fluid-extraction autoclave. The pressure was maintained at 100 bar, and the temperature was maintained above 60 °C with the constant inflow of CO_2_. The mixture of solvents and CO_2_ was vented out multiple times during the drying while withstanding the pressure and temperature. Subsequently, the aerogel was stored in a vacuum oven at 80 °C for 24 h to ensure the complete removal of the solvent. The dried sample was stored in a desiccator chamber with a relative humidity of 30% to prevent possible moisture uptake. The detailed composition of other PIR aerogels is summarized in the Supporting Information (Table S1).

### 2.6 Methods

#### 2.6.1 Chemical Characterization

The chemical structures of *p*-tolyl isocyanate and mono-/difunctional aromatic isocyanate mixtures were identified by nuclear magnetic resonance (NMR) spectroscopy conducted on a Bruker UltraShield spectrometer (400 MHz for ^1^H NMR and 100 MHz for ^13^C NMR) at 25 °C with acetone-*d*_*6*_ as solvent. The chemical composition of PIR aerogels was analyzed by Fourier transform infrared (FTIR) spectroscopy using a Thermo Fischer Scientific Nicolet iS20 spectrometer equipped with an attenuated total reflection (ATR) mode. The samples were scanned from 450 to 4000 cm^–1^.

#### 2.6.2 Physical and Structural Characterization

PIR aerogels with sample dimensions of 55 mm diameter and 10 mm thickness were used unless mentioned otherwise.

The porosity of the PIR aerogels was studied by nitrogen physisorption porosimetry. The specific surface area and pore size distribution of the aerogels were analyzed by a Brunauer–Emmett–Teller (BET) analyzer (TriStar II Plus). Before measurement, the samples were outgassed at 80 °C for 2 h under nitrogen conditions. Nitrogen (grade 5.0) and helium (grade 4.7) were chosen to measure the physisorption isotherm. The porosity and skeletal density of the PIR aerogels were measured by a helium pycnometer (AccuPyc II 1345) using helium grade 4.6. Ten data points were taken with 10 equilibrium cycles.

The morphology of PIR aerogels was characterized by scanning electron microscopy (SEM, FEI Quanta 200 3D) at an acceleration voltage of 10 kV. The aerogel samples were sputtered with gold for 40 s before testing.

The hydrophobicity of the PIR aerogels was studied by a contact angle analyzer (Data-Physics OCA30) at a relative humidity of 40%.

The water uptake test of PIR aerogels was performed by submerging the samples into a distilled water (DI water) bath for 24 h. Prior to testing, PIR aerogels with a 15 mm thickness and a 25 mm diameter were placed in an 80 °C vacuum oven for 2 h. The mass of the samples was determined before and after the samples were completely submerged under DI water for 24 h. The water uptake was calculated accordingly, in relation to the weight of the sample.

The uniaxial compression test of PIR aerogels was conducted by a ZwickRoell Materials Testing Machine, Zwicki Z2.5/TN. PIR aerogels with sample dimensions of 25 mm diameter and 15 mm thickness were used. The compressive modulus was calculated between 0.05 and 0.25% deformation ratio.

#### 2.6.3 Thermal Characterization

The thermal properties of PIR aerogels were assessed by a TGA 550 (TA Instruments) under a nitrogen atmosphere at a heating rate of 10 °C min^–1^ from 40 to 593 °C. The thermal conductivity was measured by a heat flow meter (Thermtest Inc., HFM-25) at 20 °C and 45–50% humidity according to the ASTM C518 international standard. Prior to the measurement, the machine was calibrated with EPS 1450E as reference material.

## 3 Results and Discussion

### 3.1 Design and Fabrication of PIR Aerogels

**Scheme 1:**
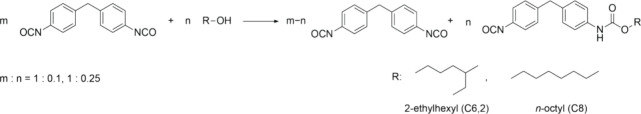
Synthesis of PIR Prepolymers from MDI and 2-Ethyl-1-hexanol (C6,2) or 1-Octanol (C8) with Different Molar Ratio (*m*:*n* = 1:0.1, 1:0.25)

In addressing the challenge of brittleness, PIR aerogels have been designed through a cocyclotrimerization process of mono- and di-isocyanates. To prepare the isocyanate mixture, MDI was reacted with either 2-ethyl-1-hexanol (C6,2) or 1-octanol (C8) in molar ratios of 1 to 0.1 or 1 to 0.25 via solvent-free synthesis (Scheme 1). Statistically, the reaction between diisocyanate and primary alcohol also could yield diurethane structures, which would remain unreactive during the gelation process.^33^ The reaction was, therefore, performed by dropwise slow-addition of the alcohol into the isocyanate at a relatively low temperature (50 °C) to increase the selectivity of isocyanate groups and to mitigate the formation of diurethane. The molar ratio of alcohols and MDI was also set to be lower than 0.25 to ensure that the subsequent reaction mixture only contains mono- and difunctional isocyanates.^33^

**Scheme 2:**
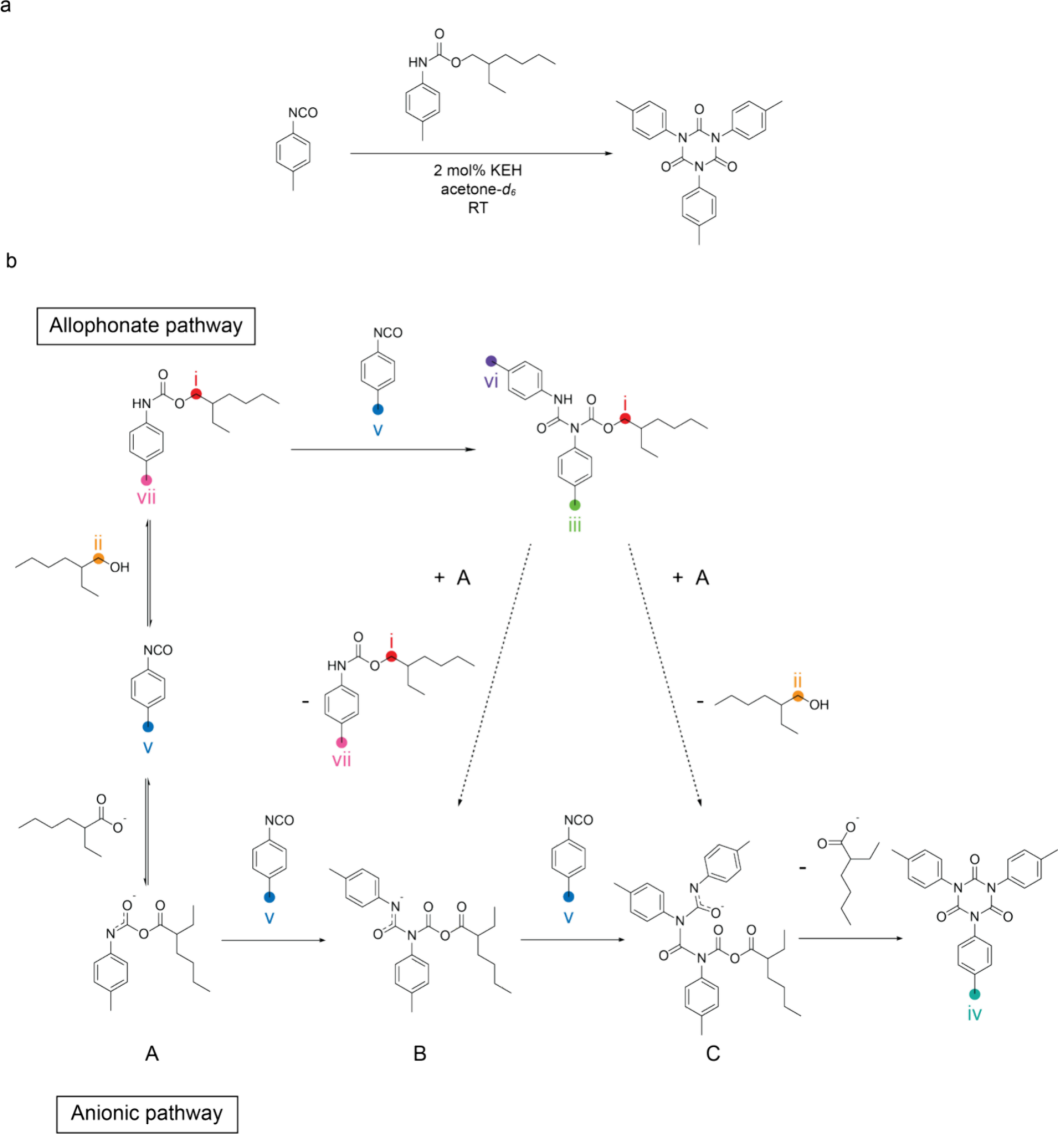
(a) Reaction Scheme between *p*-Tolyl Isocyanate and 2-Ethylhexyl *p*-Tolylcarbamate in a 1:1 Molar Ratio at Room Temperature Using 2 mol % KEH as a Catalyst; (b) Proposed Cyclotrimerization Mechanism of Isocyanates via Allophanate and Anionic Pathways

**Figure 1:**
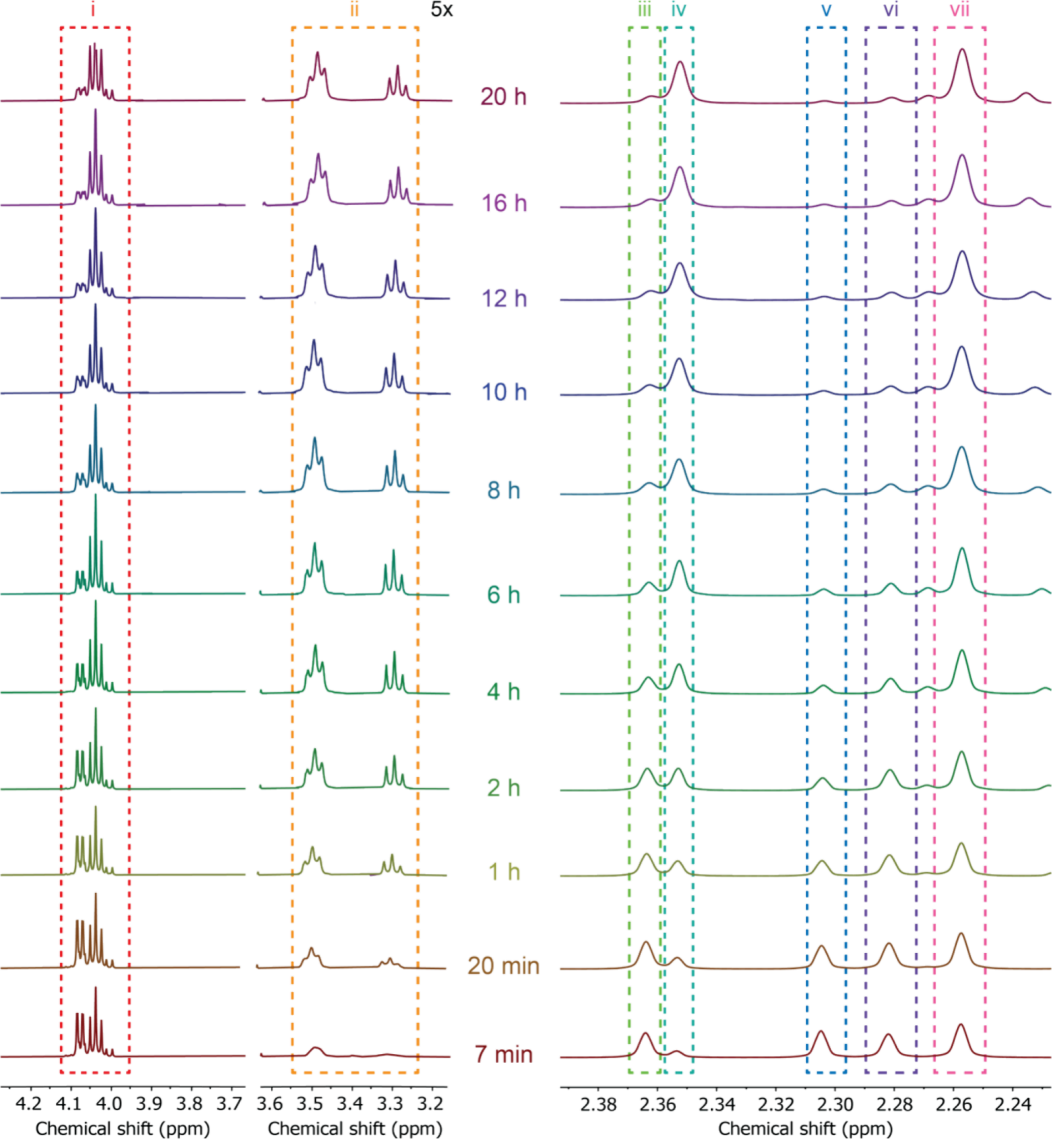
Zoomed-in image of ^1^H NMR spectra (400 MHz, acetone-*d*_*6*_) of the reaction between *p*-tolyl isocyanate and 2-ethylhexyl *p*-tolylcarbamate. The chemical shift between 2.24 and 2.38 ppm (**iii**to **vii**) and the chemical shift between 3.2 and 4.2 ppm (i and **ii**) were monitored in different time frames. The chemical shift between 3.2 and 3.6 ppm (ii) was magnified 5 times for better signal indication.

The mono- and difunctional isocyanate mixtures were further cocyclotrimerized at room temperature using potassium 2-ethylhexanoate (KEH) as a trimerization catalyst to obtain a PIR network. To study the mechanism behind the trimerization, the reaction of *p*-tolyl isocyanate and one equivalent of 2-ethylhexyl *p*-tolylcarbamate in the presence of 2 mol % KEH was carried out in an NMR tube at room temperature (Scheme 2a). According to the chemical shift of the methyl group region in ^1^H NMR spectra (δ = 2.35–2.37 ppm), the allophanate (**iii**and **vi**) was immediately formed at the onset of the reaction and gradually diminished as the isocyanurate was generated (Scheme 2b and Figure 1). In addition, the release of alcohol **ii**was also detected during the trimerization (Scheme 2b and Figure 1). These findings indicate that the trimerization mechanism follows an allophanate pathway in the presence of urethane, which aligns with the mechanism proposed by Al Nabulsi and Schwetlick^35,36^ (Figure S2). Accordingly, the isocyanate first reacts with urethane to form an allophanate as a key intermediate. Subsequently, the allophanate intermediate undergoes an addition–elimination step by reacting with a nucleophilic species and releasing the alcohol (due to the equivalent ratio of *p*-tolyl isocyanate and 2-ethylhexyl *p*-tolylcarbamate), resulting in the formation of the isocyanurate structure. Furthermore, almost full consumption of allophanate was observed, and a near quantitative yield of isocyanaurate was obtained. Therefore, KEH can be regarded as an effective catalyst.^30^

### 3.2 Preparation and Chemical Characterization of PIR Aerogels

**Scheme 3:**
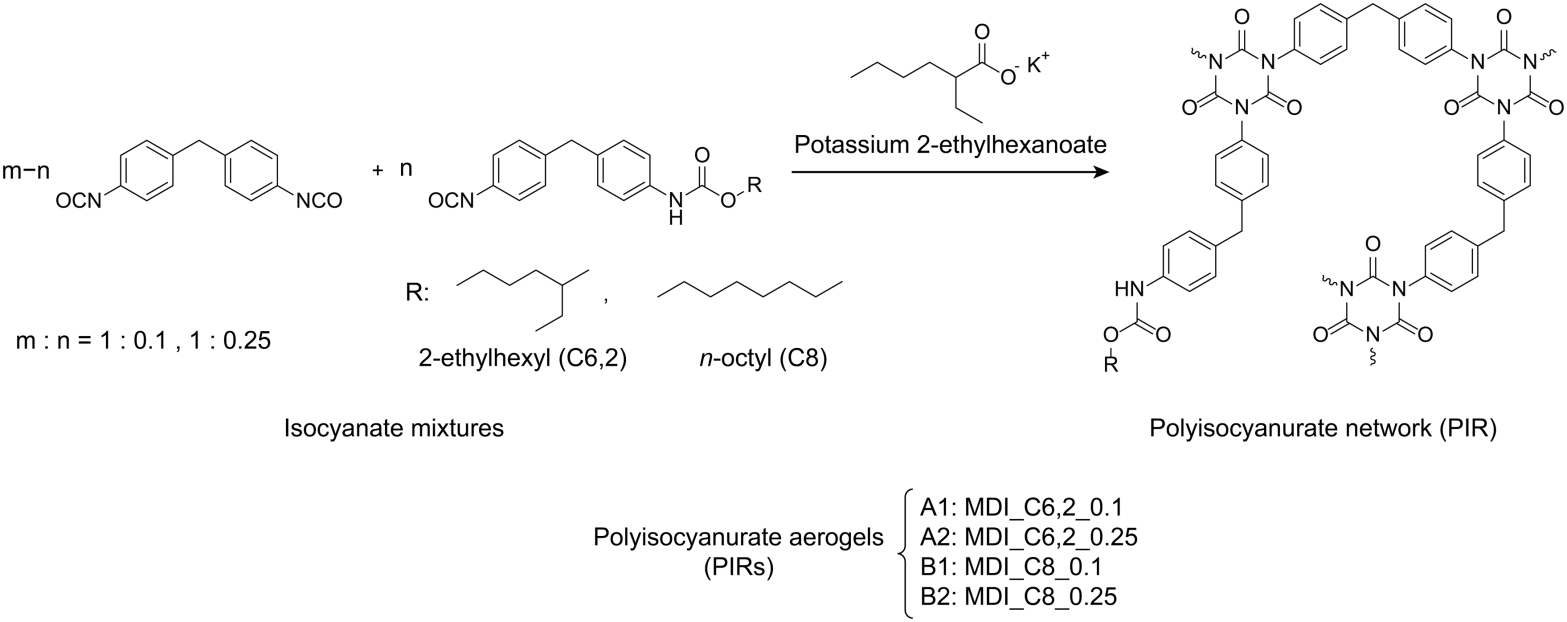
Reaction Scheme Illustrating the Trimerization Reaction of Isocyanate Mixtures Using Potassium 2-Ethyl-1-hexonate (KEH) as Catalyst Four sets of PIR aerogels were made based on different isocyanate mixtures, namely, **PIR-A1**, **PIR-A2**, **PIR-B1**, and **PIR-B2.**

**Figure 2:**
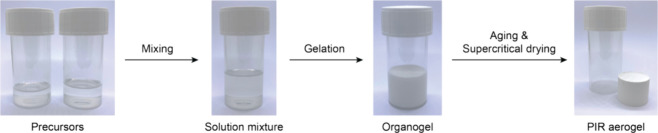
Schematic representation of the aerogel synthesis protocol. Precursors (isocyanates and catalyst) were dissolved separately in 3-pentanone, mixed at room temperature, to obtain stable organogels. After curing for 24 h at ambient condition, the organogels were supercritically dried with CO_2_ to yield PIR aerogels.

The PIR aerogels were further prepared by using the synthesized isocyanate mixtures. As described in the previous section, four sets of different isocyanate mixtures were synthesized by using different primary alcohol inputs and alcohol/isocyanate molar ratios (Scheme 3). The cocyclotrimerization of the isocyanate mixtures was initiated in 3-pentanone as the solvent and with KEH as the catalyst at room temperature. Opaque and stable organogels were formed after 2 to 8 h. According to the mechanism study, the reaction would be completed in 20 h (Figure 1), thus the organogels were left at room temperature for 24 h to ensure high conversion to isocyanurates. Subsequently, the organogels were dried by supercritical CO_2_ drying to obtain an array of PIR aerogels (Figure 2). To study the effect of the cross-linking density and alkyl chain moiety of PIR aerogels, a reference aerogel, namely, **PIR-R1**, prepared from trimerization of neat MDI, was also included (Table S1).

The chemical composition of PIR aerogels was investigated by FTIR. As shown in Figure S3, the characteristic stretching vibration of isocyanates (−N=C=O) could not be found in all of the PIR aerogels (2275 cm^–1^), suggesting a full consumption of isocyanate groups. Moreover, the characteristic peaks of isocyanurate C–N stretching were also observed at 1474–1338 cm^–1^, indicating the formation of PIR during gelation.

### 3.3 Physical Properties of PIR Aerogels

The physical properties of PIR aerogels, including the material density, porosity, and hydrophobicity, are summarized in Table 1. PIR aerogels with the 2-ethylhexyl (C6,2) moiety showed larger linear shrinkage after supercritical drying compared to those with *n*-octyl (C8) chains and the reference aerogel. This result ultimately led to higher density (>200 mg cm^–3^) and lower porosity (<80%), which is not appealing to aerogel properties. With C8 side chains, both **PIR-B1** and **PIR-B2** exhibited low bulk density (<180 mg cm^–3^) and significantly high porosity of ∼85%, which is a prerequisite for creating ultralight materials.^3^

**Table 1:** General Material Properties of PIR Aerogels

name	composition	bulk density ρ_b_ [mg cm^–3^]	linear shrinkage [%]^a^	skeletal density ρ_s_ [g cm^–3^]	porosity Π [%]^b^	contact angle [°]	water uptake [%]^c^
**PIR-R1**	MDI	205	9.6	1.23	83	n.a.^d^	471
**PIR-A1**	MDI_C6,2_0.1	219	11.4	1.21	82	n.a.^d^	437
**PIR-A2**	MDI_C6,2_0.25	263	18.4	1.23	79	105	12
**PIR-B1**	MDI_C8_0.1	179	6.0	1.26	86	n.a.^d^	255
**PIR-B2**	MDI_C8_0.25	161	5.2	1.29	88	107	7

a Calculated based on the diameter change of the monolith.

b Porosity was calculated via equation: Π = (1 – ρ_b_/ρ_s_) × 100%.

c Water uptake ratio was recorded as specimens weight difference after submerging in DI water for 24 h.

d The sample completely absorbed the water droplets.

For long-term applications of aerogels, hydrophobicity is one of the most crucial properties. Thus, rigorous water uptake tests were conducted to determine the water repellence of the PIR aerogels by immersing them in DI water for 24 h. **PIR-R1** showed the highest water uptake value due to the absence of hydrophobic side chains. Similarly, **PIR-A1** and **PIR-B1** exhibited significant water uptake, exceeding two-four times their initial weights. This indicates that a low alkyl chain content does not impart hydrophobic characteristics to PIR aerogels. Conversely, aerogels with a higher alkyl chain moiety content, such as **PIR-A2** and **PIR-B2**, demonstrated enhanced inherent hydrophobicity, as evidenced by their water uptake values being more than 20 times lower compared to those with lower alkyl chain contents. Additionally, the hydrophobicity was evaluated by water contact angle measurements. **PIR-R1**, **PIR-A1**, and **PIR-B1** samples all absorbed water completely, whereas the contact angle values of **PIR-A2** and **PIR-B2** were 105° and 107°, respectively (Figure S4). These results align with the water uptake ratios, indicating that a higher alkyl chain content is necessary for inherent hydrophobicity.

### 3.4 Microstructure of PIR Aerogels

**Figure 3:**
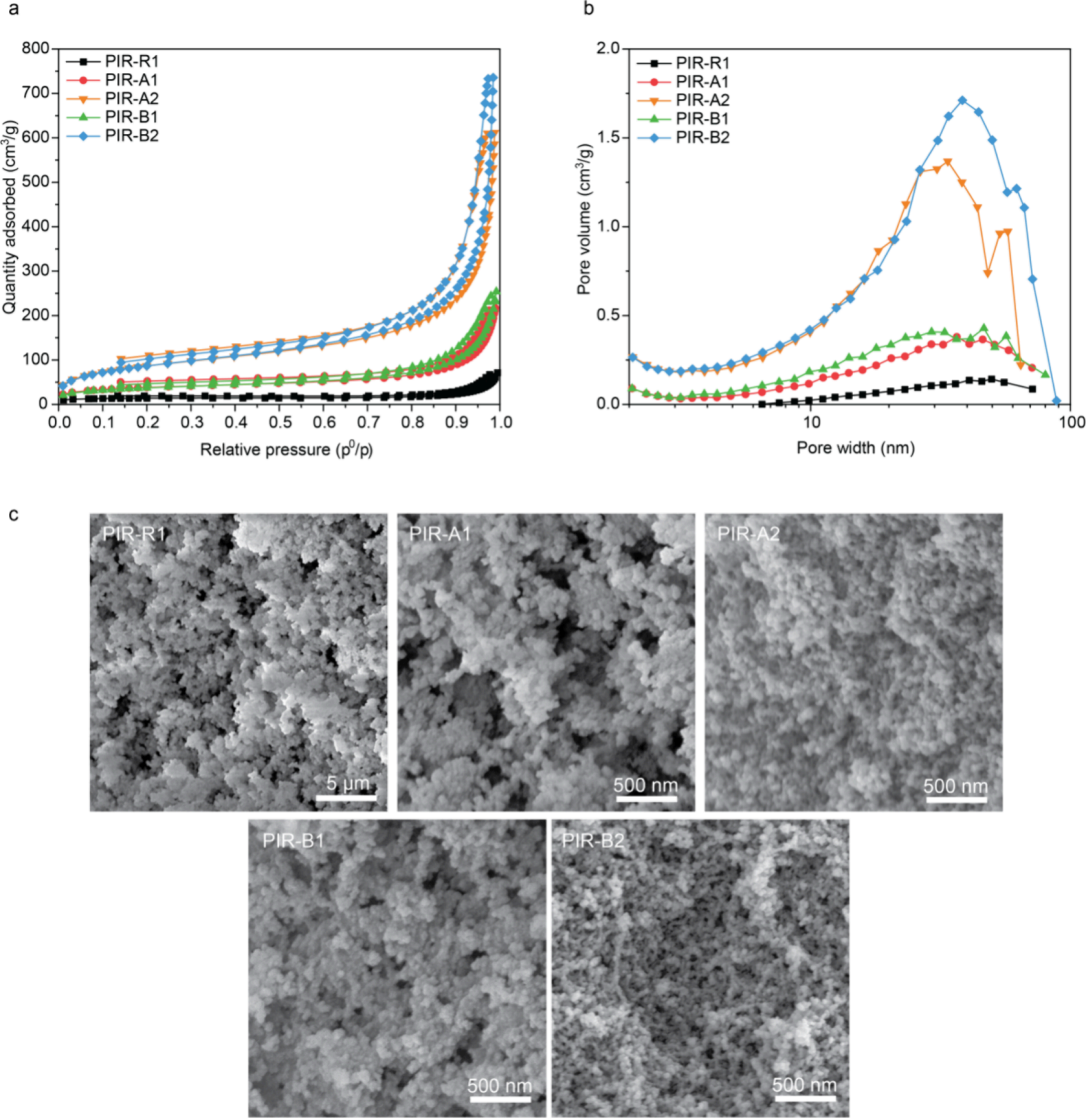
(a) N_2_ adsorption and desorption isotherms of PIR aerogels at 77 K. (b) BJH pore size distribution of PIR aerogels. (c) SEM micrographs of PIR aerogels.

The microstructures of PIR aerogels were investigated using nitrogen sorption porosimetry. According to the IUPAC classification, all the isotherms of PIR aerogels displayed Type IV characteristics, where the hysteresis in the desorption isotherm can be seen around the region *p*/*p*^0^ > 0.7, indicating a wide range of mesopore distributions. Upon comparison of the isotherms of different PIR aerogels, it was observed that **PIR-A2** and **PIR-B2** exhibited higher nitrogen adsorbed values than **PIR-A1**, **PIR-B1**, and **PIR-R1**, suggesting a larger mesopore volume (Figure 3a). Additionally, **PIR-A2** and **PIR-B2** demonstrated notably higher specific surface areas (Table 2), indicating that the incorporation of alkyl side chains positively impacts the increase in specific surface area values. The Barrett–Joyner–Halenda (BJH) analysis revealed a mesopore size distribution ranging from 20 to 70 nm with relatively high pore volume (Figure 3b).

**Table 2:** Microstructure Properties of PIR Aerogels

name	composition	specific surface area [m^2^ g^–1^]	pore volume [cm^3^ g^–1^]
**PIR-R1**	MDI	45	0.08
**PIR-A1**	MDI_C6,2_0.1	146	0.37
**PIR-A2**	MDI_C6,2_0.25	309	0.91
**PIR-B1**	MDI_C8_0.1	192	0.40
**PIR-B2**	MDI_C8_0.25	311	1.13

The morphology of PIR aerogels was further examined by SEM (Figures 3c and S5). The SEM images reveal isotropic molecular structures, showcasing a broad range of porous networks with bead-like structures. **PIR-R1** exhibited morphology with larger solid particle clusters, while **PIR-A1** and **PIR-B1** formed slightly smaller particle aggregates. On the other hand, **PIR-A2** and **PIR-B2** showed smaller spherical solid networks. This trend aligns well with nitrogen porosimetry data, where **PIR-A2** and **PIR-B2** gave higher specific surface areas and mesopore volumes. It is noteworthy that the morphology of the PIR network can be influenced by various factors such as catalyst concentrations^37^ and solvent systems.^25^ Herein, we found out that the incorporation of monoalkyl chains significantly impacted the morphology of resulting PIR aerogels.

### 3.5 Thermal and Mechanical Properties of PIR Aerogels

Owing to their nanoscale architectures and related Knudsen effect,^6^ PIR aerogels are anticipated to demonstrate exceptional thermal insulation performance. The thermal conductivities of the PIR aerogels were measured using a heat flow meter, following the ASTM C518 standard (Table 3). **PIR-R1**, with its low specific surface area and mesopore volume, exhibited the highest thermal conductivity of 0.029 W m^–1^ K^–1^, surpassing that of still air. **PIR-A1** and **PIR-B1** had slightly lower thermal conductivities, potentially attributed to the increase in the specific surface area and pore volume. Notably, **PIR-B2** had the lowest value at 0.017 W m^–1^ K^–1^, while **PIR-A2** showed a slightly higher value of 0.019 W m^–1^ K^–1^. Despite having a high specific surface area and mesopore volume, **PIR-A2** also had a higher bulk density than **PIR-B2**, resulting in a higher solid thermal conductivity and overall thermal conductivity. Overall, **PIR-B2** showed a lower thermal conductivity value than other reported organic aerogels (Figure S6). The low thermal conductivity value of **PIR-B2** is derived from the synergistic combination of its low bulk density and high specific surface area, which not only minimizes solid phase conductivity but also hinders gas thermal conduction through the effective Knudsen effect.^6^ In addition, **PIR-B2** exhibited nearly the same insulation performance level as the commercial polyurea aerogels prepared according to the patented example.^38,39^

**Table 3:** Thermal Properties of PIR Aerogels

name	composition	thermal conductivity (W m^–^^1^ K^–1^)^a^	*T*_d5%_ (°C)^b^	char formation (%)^c^
polyurea aerogel^d^		0.0179	213	25.6
**PIR-R1**	MDI	0.0294	267	47.5
**PIR-A1**	MDI_C6,2_0.1	0.0248	242	49.2
**PIR-A2**	MDI_C6,2_0.25	0.0196	238	47.1
**PIR-B1**	MDI_C8_0.1	0.0271	241	44.5
**PIR-B2**	MDI_C8_0.25	0.0168	246	46.1

a Thermal conductivities were measured with a heat flow meter (Thermtest Inc., HFM-25).

b Decomposition temperatures for 5% weight loss.

c Char formation yield at 593 °C measured by TGA.

d Reference polyurea aerogel was prepared according to patent.^38,39^

**Figure 4:**
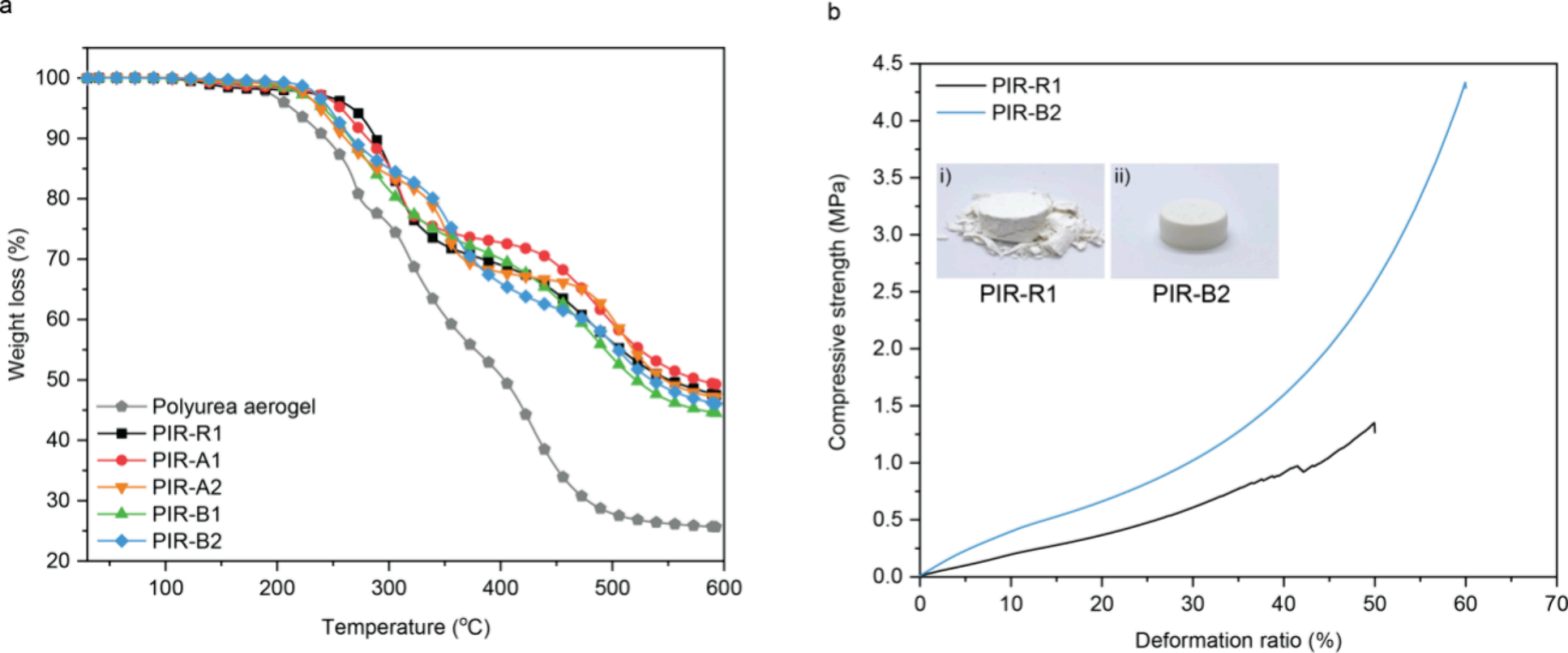
(a) TGA curves of PIR aerogels and polyurea aerogel, ramping from 40 to 593 °C with a heating rate of 10 °C/min. (b) Compression–deformation curves of **PIR-R1** and **PIR-B2**. Photographs of (i) **PIR-R1** and (ii) **PIR-B2** after compression testing.

The thermal stability of the PIR aerogels was assessed with thermogravimetric analysis (TGA) and compared to the commercial polyurea aerogel. The decomposition temperature of polyurea aerogels at 5% weight loss (*T*_d5%_) was 213 °C (Figure 4a). Upon the incorporation of PIR, the *T*_d5%_ value of **PIR-R1** increased significantly to 267 °C. However, the *T*_d5%_ values of **PIR-A** and **PIR-B** pairs slightly decreased due to the higher content of alkyl side chains in the material. Nevertheless, due to the presence of PIR structures, the char formation of PIR aerogels was notably higher than that of polyurea aerogels at 593 °C, which validates superior thermal stability than conventional aerogel materials.

Based on the previous discussion, **PIR-B2** has shown the most favorable properties regarding bulk density and porosity. Hence, **PIR-B2** was chosen to test its mechanical properties. Uniaxial compression tests were conducted on **PIR-R1** and **PIR-B2** to evaluate the mechanical performance (Figure 4b). **PIR-B2** tolerated high compressive strains without any cracks above a 60% deformation ratio. In contrast, **PIR-R1** formed cracks and blisters at 35% deformation ratio, suggesting the brittleness of PIRs. Furthermore, **PIR-B2** exhibited a high compressive modulus of 4.45 MPa, whereas **PIR-R1** showed a lower compressive modulus of 1.80 MPa. A detailed comparison of **PIR-B2** with other organic aerogels in terms of compressive modulus can be found in the Supporting Information (Figure S7). In general, the mechanical performance improvement observed in **PIR-B2** validates that the combination of cocyclotrimerization of mono-/difunctional isocyanates and the incorporation of alkyl side chains can effectively reduce the brittleness of the PIR structure.

## 4 Conclusions

Herein, we successfully prepared a library of high-performance organic PIR aerogels via cocyclotrimerization of di- and monofunctional isocyanates decorated with long alkyl chains. The resulting PIR aerogels exhibited low bulk density, high porosity (>89%), a large specific surface area (∼300 m^2^ g^–1^), and ultralow thermal conductivity (∼0.017 W m^–1^ K^–1^). Furthermore, the incorporation of an alkyl side chain granted these PIR aerogels intrinsic hydrophobicity without the need for postmodification. The cocylotrimerization of mono-/difunctional isocyanates also led to high PIR conversion. Due to the high content of PIR, these aerogels exhibited great thermal stability (*T*_d5%_ > 240 °C) and good char formation at 593 °C (>46%). Notably, their mechanical strength was significantly enhanced as compared with reference aerogel, as evidenced by the enhancement of compression modulus from 1.8 to 4.5 MPa. These high-performance PIR aerogels hold significant promise for a wide range of insulation applications.

## Supporting Information Available

Synthesis of isocyanate mixtures; synthesis of the reference polyurea aerogel; supercritical CO_2_ drying setup; ^1^H NMR characterization of monomers; FTIR characterization of PIR aerogels; water contact angle of PIR aerogels; SEM micrograph of **PIR-R1**; and comparison graphs of **PIR-B2** and other organic aerogels in terms of thermal conductivity and compressive modulus (PDF)
