# Supplementary material for: Fabrication of High-Performance Polyisocyanurate Aerogels through Cocyclotrimerization of 4,4′-Methylene Diphenyl Diisocyanate and Its Mono-Urethane Derivatives
Source: ACS Appl Mater Interfaces. 2024 Jun 26;16(27):35604–12. doi: 10.1021/acsami.4c07480 (PMC11247422; doi:10.1021/acsami.4c07480)
Supplement: Supplementary file 1 — am4c07480_si_001.pdf [file am4c07480_si_001.pdf]

Supporting Information

Fabrication of High-performance Polyisocyanurate Aerogels through Co-cyclotrimerization of 4,4'-Methylene Diphenyl Diisocyanate and its Mono Urethane Derivatives

*Changlin Wang,[a] Yunfei Guo,[a] Tankut Türel,[a] and Željko Tomović[a]**

[a] Polymer Performance Materials Group, Department of Chemical Engineering and Chemistry, Eindhoven University of Technology, 5600 MB Eindhoven, The Netherlands.

Corresponding Authors

Željko Tomović

E-mail: z.tomovic@tue.nl

Table of Contents

Synthesis of isocyanate mixture – MDI_C6,2_0.1 3

Synthesis of isocyanate mixture – MDI_C6,2_0.25 3

Synthesis of isocyanate mixture – MDI_C8_0.1 3

Synthesis of isocyanate mixture – MDI_C8_0.25 3

Synthesis of the reference polyurea aerogel 3

Supercritical CO_2 drying setup 4

Supplementary tables and figures 5

Synthesis of isocyanate mixture – MDI_C6,2_0.1

4,4'-MDI (87.6 g, 0.35 mol) was introduced into a dry 3-neck flask equipped with a dropping funnel and stirred under Ar atmosphere at 50 °C. 2-ethyl-1-hexanol (4.56 g, 35 mmol) was added dropwise while keeping the internal temperature under 55 °C. Upon complete addition, the reaction was immediately stopped and the mixture was collected and stored at −20 °C. The product was confirmed by ^{1}H NMR (Figure S8).

Synthesis of isocyanate mixture – MDI_C6,2_0.25

4,4'-MDI (87.6 g, 0.35 mol) was introduced into a dry 3-neck flask equipped with a dropping funnel and stirred under Ar atmosphere at 50 °C. 2-ethyl-1-hexanol (11.4 g, 87.5 mmol) was added dropwise while keeping the internal temperature under 55 °C. Upon complete addition, the reaction was immediately stopped and the mixture was collected and stored at −20 °C. The product was confirmed by ^{1}H NMR (Figure S9).

Synthesis of isocyanate mixture – MDI_C8_0.1

4,4'-MDI (87.6 g, 0.35 mol) was introduced into a dry 3-neck flask equipped with a dropping funnel and stirred under Ar atmosphere at 50 °C. 1-octanol (4.56 g, 35 mmol) was added dropwise while keeping internal temperature under 55 °C. Upon complete addition, the reaction was immediately stopped and the mixture was collected and stored at −20 °C. The product was confirmed by ^{1}H NMR (Figure S10).

Synthesis of isocyanate mixture – MDI_C8_0.25

4,4'-MDI (87.6 g, 0.35 mol) was introduced into in a dry 3-neck flask equipped with a dropping funnel and stirred under Ar atmosphere at 50 °C. 1-octanol (11.4 g, 87.5 mmol) was added dropwise while keeping internal temperature under 55 °C. Upon complete addition, the reaction was immediately stopped and the mixture was collected and stored at −20 °C. The product was confirmed by ^{1}H NMR (Figure S11).

Synthesis of the reference polyurea aerogel

The reference polyurea aerogel was prepared according to a BASF patent, example 8.[1,2] The organogel was produced by mixing component A and B. Component A consisted of 2.95 g M200 dissolved in 13.5 g MEK, while component B comprised 0.74 g MDEA, 0.25 g Dabco® K-15, 0.25 g *n*-butanol, and 0.25 g water dissolved in 13.5 g MEK. The gelling was initiated by mixing two components into one vial. The mixture was stirred to obtain a homogeneous solution. The solution was then poured into a PTFE mold with 65 mm diameter and placed in

the fume hood for 24 h under ambient conditions to age. Subsequently, the organogel was transferred to an autoclave, submerged in acetone, and sealed in a supercritical fluid-extraction autoclave. The pressure was maintained at 100 bar with the temperature kept above 60 °C through continuous inflow of CO_2. Throughout the drying process, the mixture of solvent and CO_2 was vented out multiple times while sustaining pressure and temperature conditions. The resulting aerogels were then stored in a nitrogen oven at 80 °C for 24 h to ensure complete removal of the solvent. Finally, the dried sample was stored in a desiccator chamber with relative humidity of 30% to prevent possible moisture uptake.

Supercritical CO_2 drying setup

For the supercritical drying process (SCD), Liquid CO_2 grade 2.7 (purity >99.7%) serves as the exchange agent. The drying setup utilizes the high-pressure extraction/drying units "HP-DE200," comprising an autoclave (Scheme S1) provided by Eurotechnica. The autoclave has a maximum working temperature of 100 °C and can operate under pressures up to 220 bar. Equipped with a thermowell containing a NiCr-Ni thermocouple, the autoclave monitors internal temperature. Additionally, two venting tubes are attached for solvent extraction and depressurization. The supercritical drying system includes two thermal baths (Selecta, UNITRONIC 200) for heat exchangers 1 and 2 (4 and 7), a mechanical pump (5) provided by Maximator, a check number valve (2), five needle valves (3, 6, 7, 8, 10, and 11), and a CO2 bottle (1) (Scheme S1).

The process begins by introducing gels into the autoclave (9) and covering them with the solvent used for gel formation to prevent premature solvent evaporation and minimize shrinkage. CO_2 pressure is gradually increased up to 100 bar to extract solvent from the wet gel pores. Heat exchanger 2 (7) maintains the autoclave at a constant temperature of 60 °C. Once supercritical conditions are reached, solvent extraction from the gels in the autoclave (9) occurs. Supercritical CO_2 enriched with extracted solvent is vented out by releasing the autoclave, with pressure maintained by constant fresh CO_2 input. Venting lasts approximately 10 to 15 minutes before the autoclave is closed for further extraction. Three cycles of extraction are conducted with a waiting interval of 30 minutes each. Finally, when aerogel pores are free of solvent, pressure is slowly released to atmospheric pressure through the metering valve for 45 minutes.

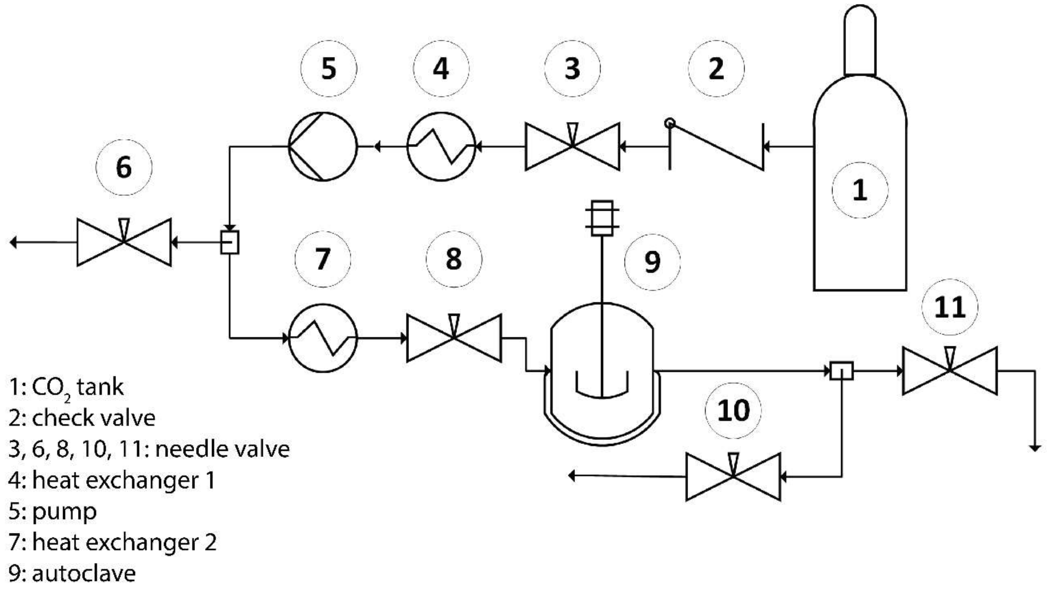

Scheme S1. Flow diagram of supercritical drying setup.

Supplementary tables and figures

Table S1. Composition of PIR aerogels

Name	MDI [g]	MDI-C6,2_0.1 [g]	MDI-C6,2_0.25 [g]	MDI-C8_0.1 [g]	MDI-C8_0.25 [g]	KEH [g]	3-pentanone [g]
PIR-R1	4.98	-	-	-	-	0.098	27
PIR-A1	-	4.90	-	-	-	0.098	27
PIR-A2	-	-	4.90	-	-	0.098	27
PIR-B1	-	-	-	4.99	-	0.012	27
PIR-B2	-	-	-	-	4.99	0.012	27
PIR-R1[a]	1.64	-	-	-	-	0.032	9
PIR-B2[a]	-	-	-	-	1.66	0.004	9

[a]The samples were prepared for compression testing.

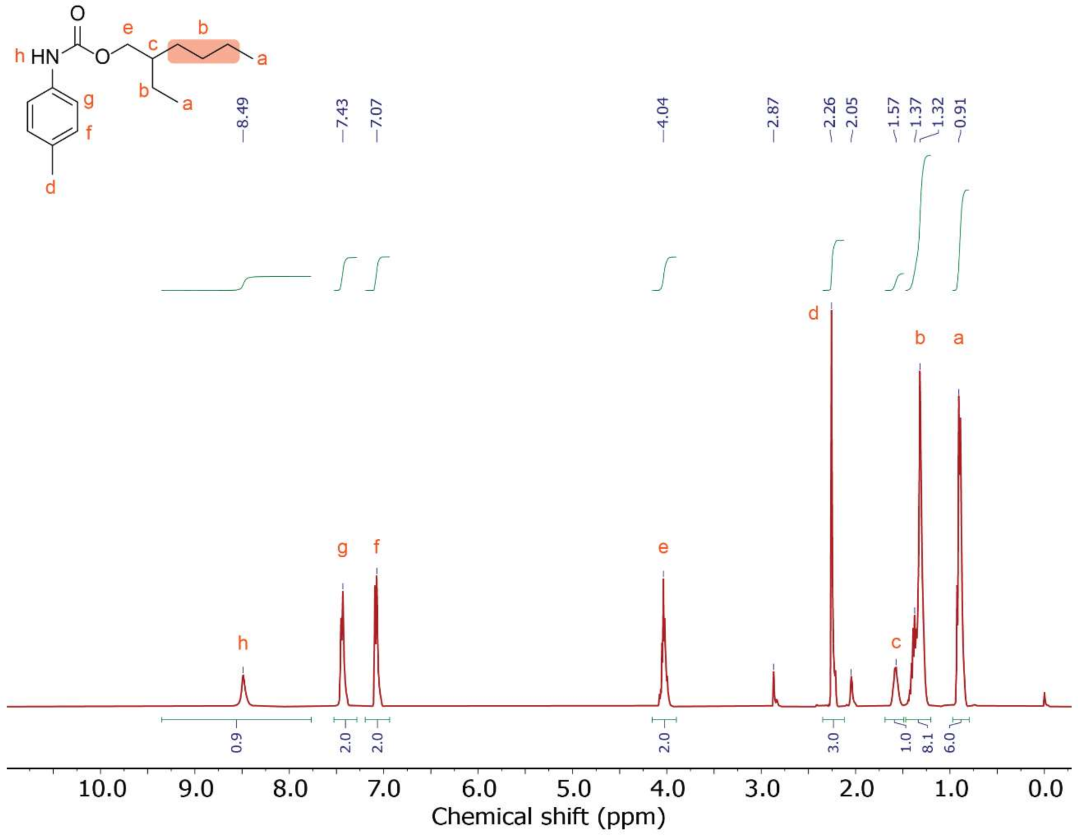

Figure S1. ^{1}H NMR spectrum (400 MHz, acetone-$_{d6}$) of 2-ethylhexyl *p*-tolylcarbamate.

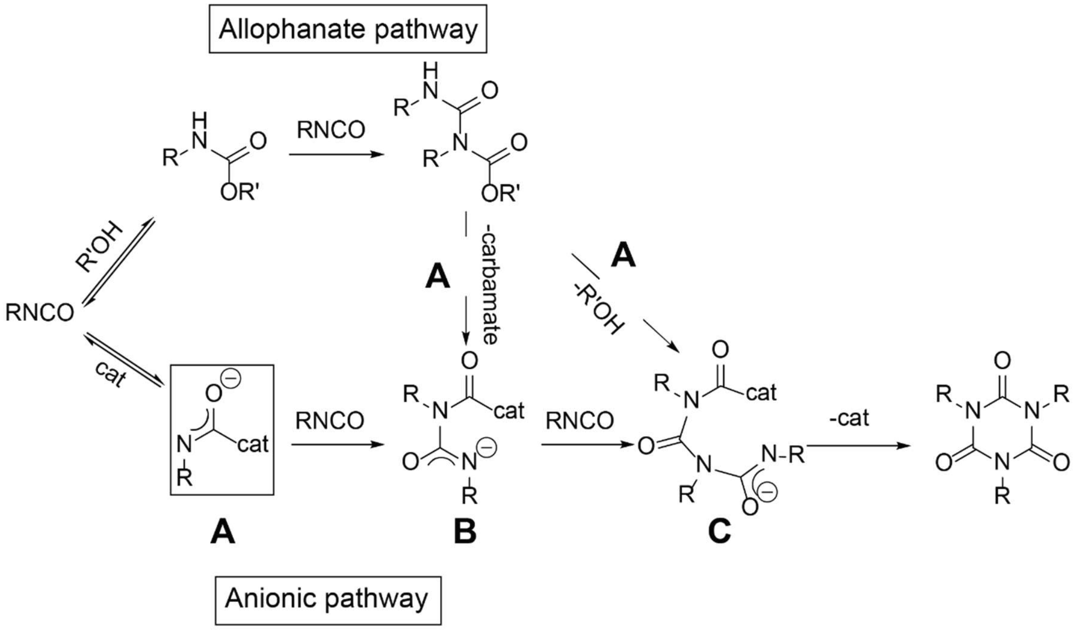

Figure S2. Proposed cyclotrimerization mechanism of isocyanates via allophanate and anionic pathways adapted from reference.[3]

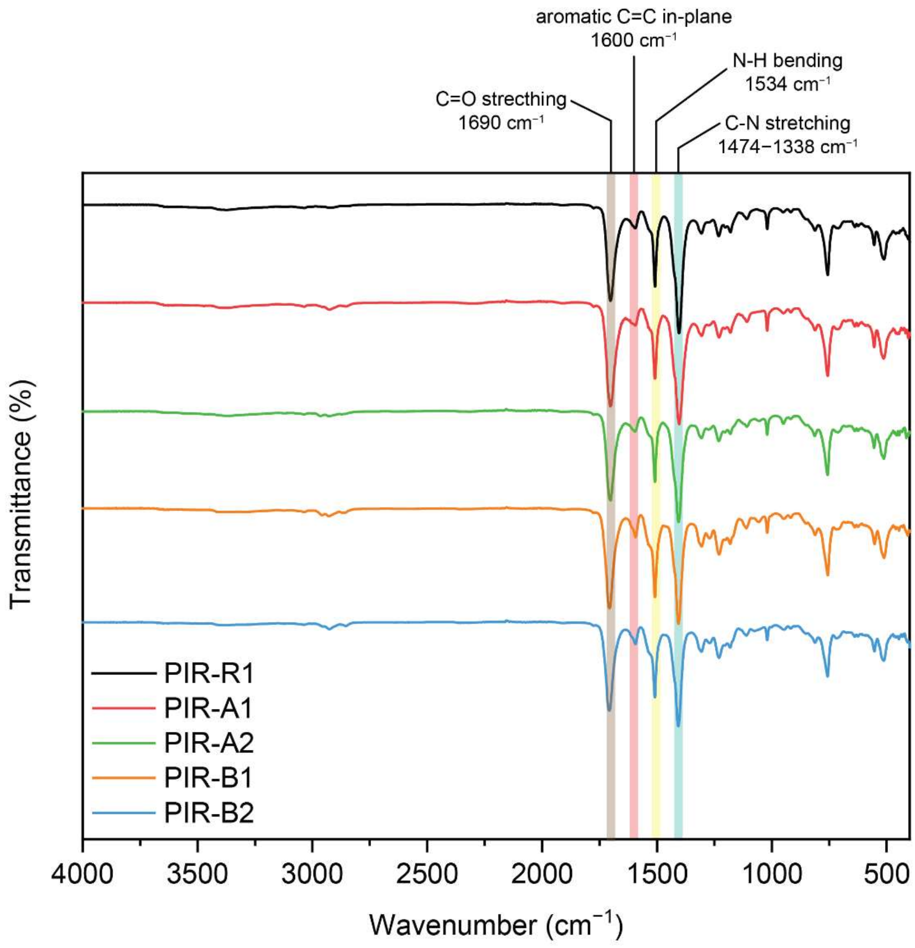

Figure S3. FT-IR spectra of PIR aerogels

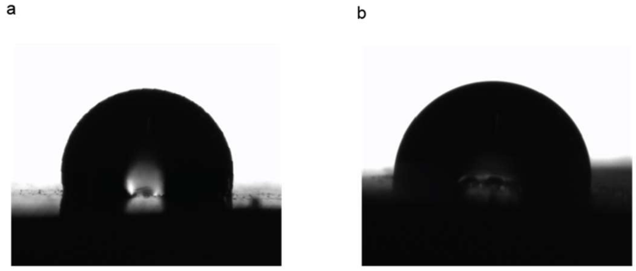

Figure S4. Water contact angle of a) **PIR-A2** and b) **PIR B2.**

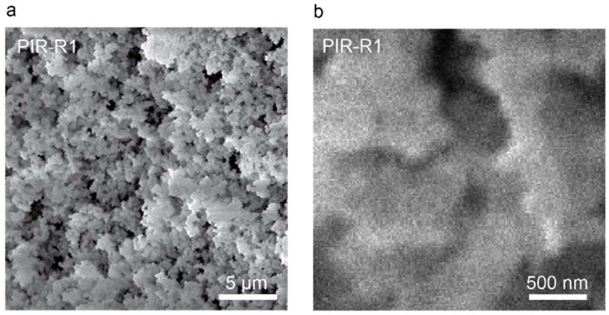

Figure S5. SEM micrographs of PIR-R1. a) 10,000x magnification, b) 100,000x magnification.

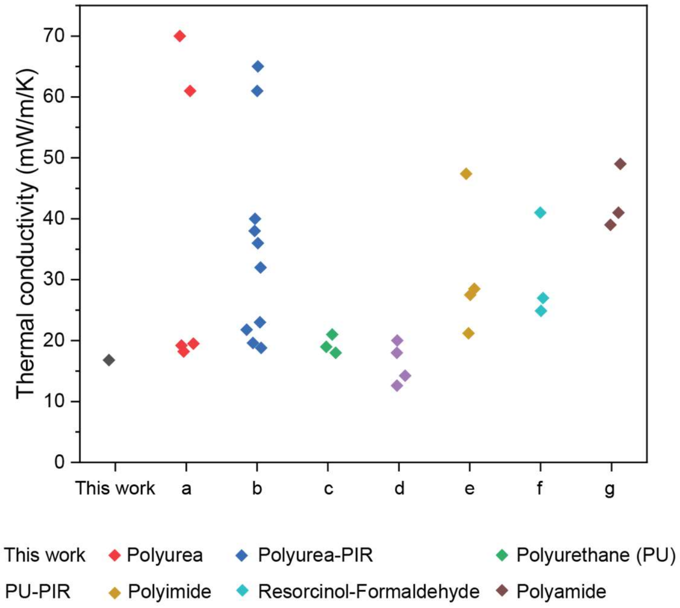

Figure S6. Comparison of thermal conductivity of **PIR-B2** aerogel with other organic aerogels, including (a) polyurea aerogels[4,5], (b) Polyurea-polyisocyanurate (PIR) aerogels[6,7], (c) Polyurethane (PU) aerogels[8], (d) PU-PIR aerogels[9,10], (e) polyimide-based aerogels[11–14], (f) resorcinol-formaldehyde (RF) aerogels[15,16], and (g) polyamide aerogels[17].

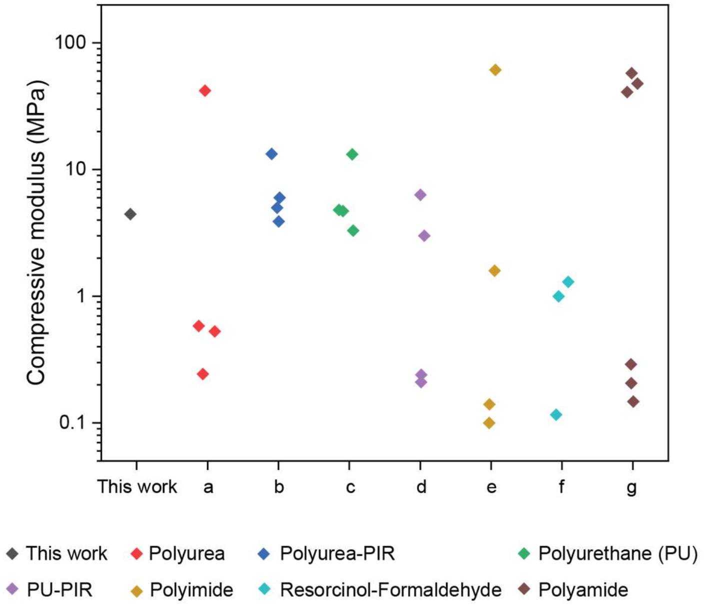

Figure S7. Comparison of compressive modulus of **PIR-B2** aerogel with other organic aerogels, including (a) polyurea aerogels[4,5], (b) Polyurea-polyisocyanurate (PIR) aerogels[6,7], (c) Polyurethane (PU) aerogels[8], (d) PU-PIR aerogels[9,10], (e) polyimide-based aerogels[11–14], (f) resorcinol-formaldehyde (RF) aerogels[15,16], and (g) polyamide aerogels[17,18].

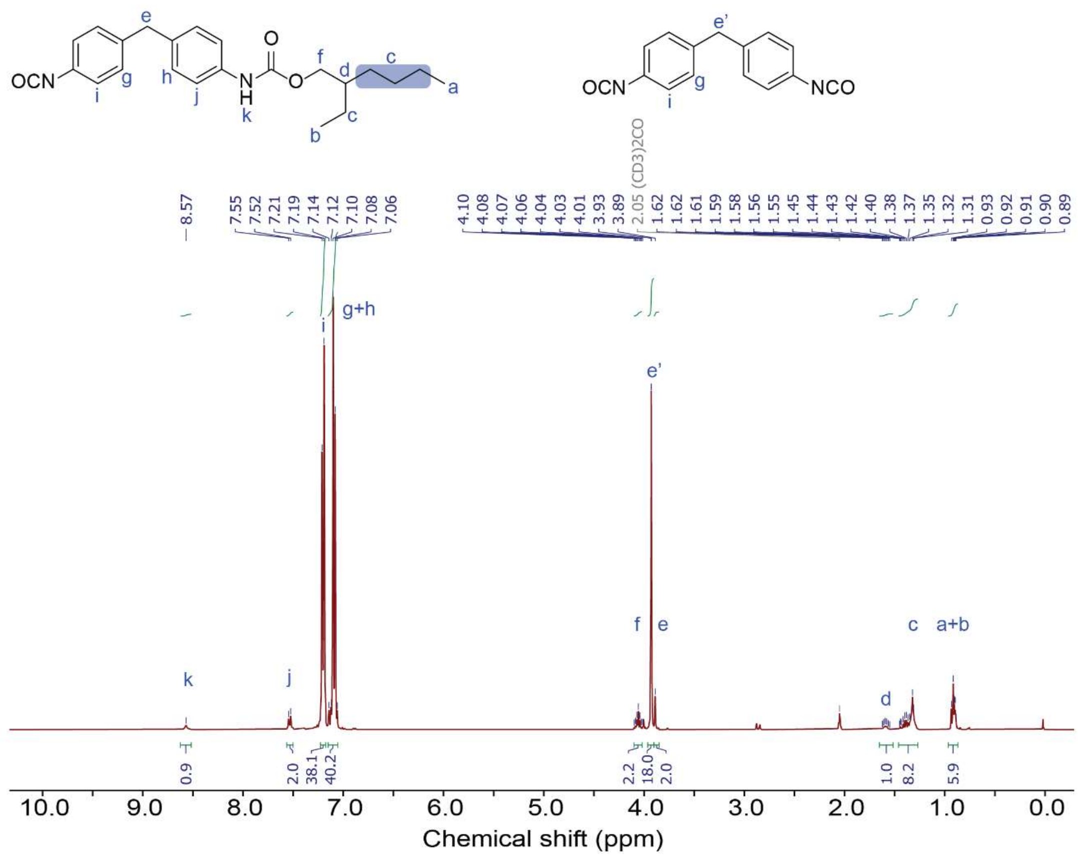

Figure S8. ^{1}H NMR spectrum (400 MHz, acetone-$_{d6}$) of MDI_C6,2_1_0.1

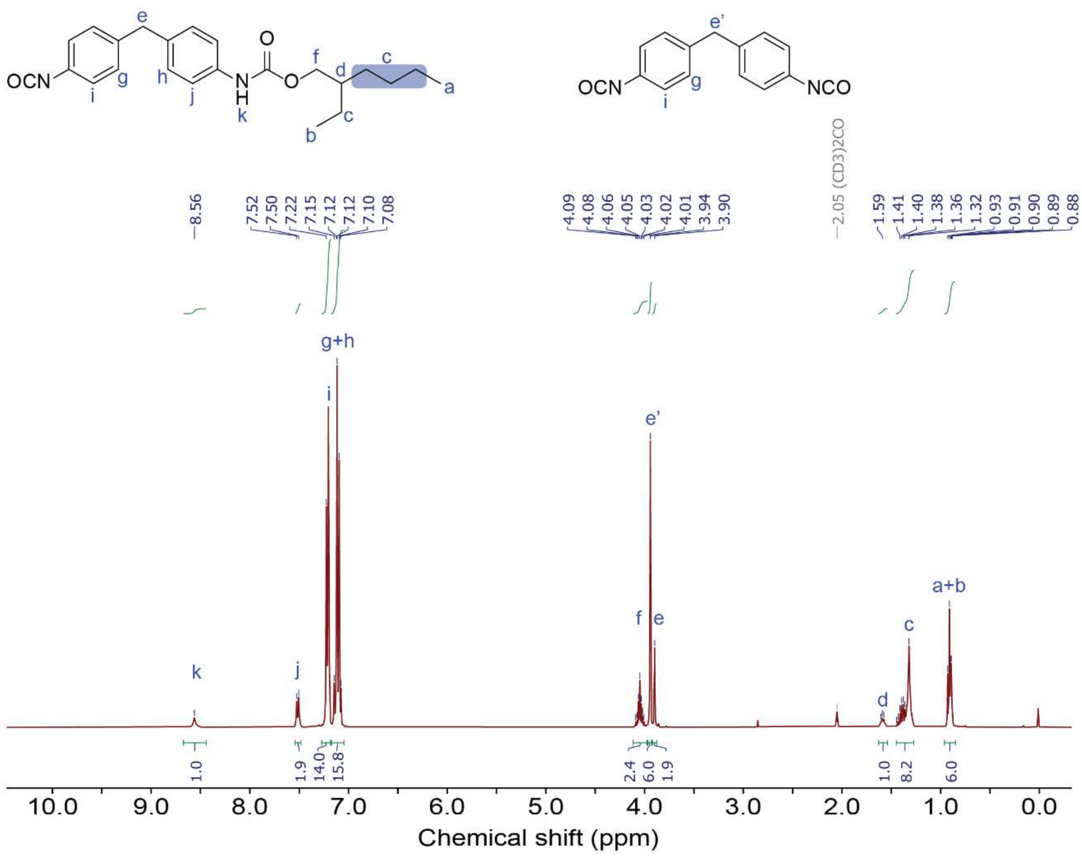

Figure S9. ^{1}H NMR spectrum(400 MHz, acetone-$_{d6}$) of MDI_C6,2_1_0.25

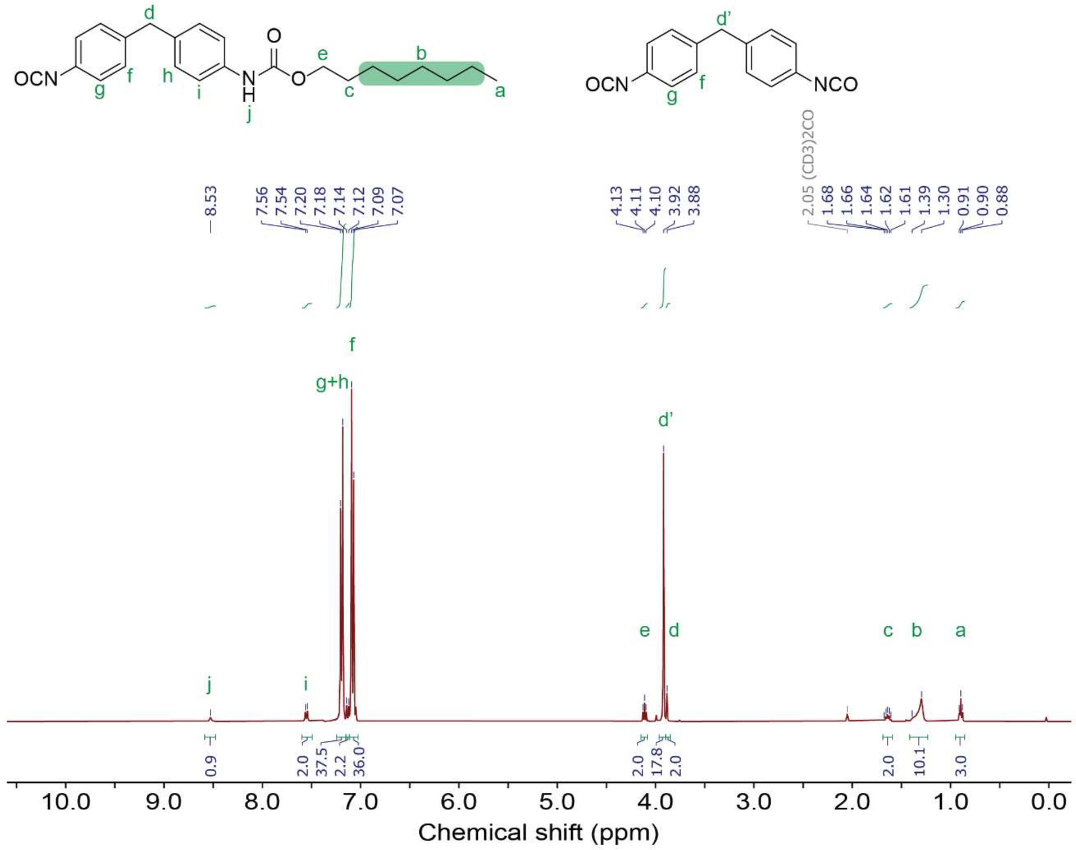

Figure S10. ^{1}H NMR spectrum (400 MHz, acetone-$_{d6}$) of MDI_C8_1_0.1

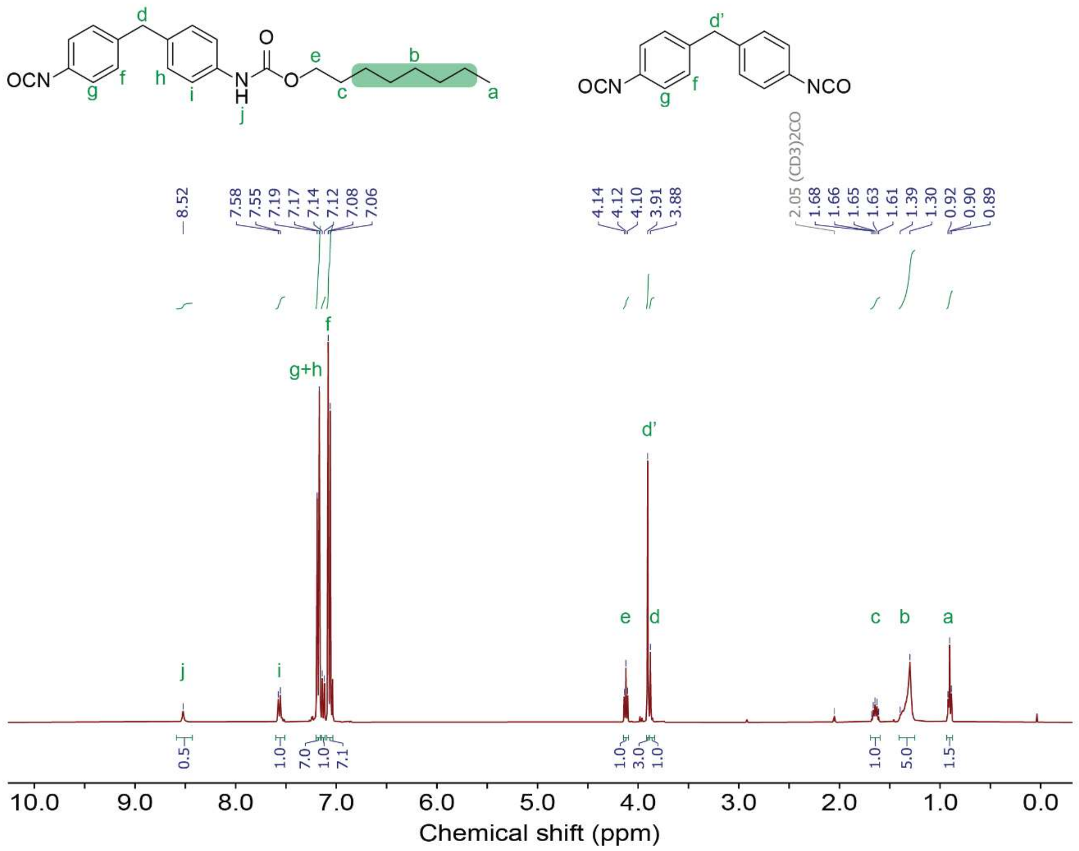

Figure S11. ^{1}H NMR spectrum (400 MHz, acetone-$_{d6}$) of MDI_C8_1_0.25

References

(1) Fricke, M., Weinrich, D., Process for producing porous materials WO 2,015,144,675 A1, 2015.

(2) Fricke, M., Weinrich, D., *SLENTITE® PU aerogels aerogel-it*. https://www.aerogel-it.de/slentite-and-pu-aerogels (Accessed in March 2024).

(3) Guo, Y.; Muuronen, M.; Lucas, F.; Sijbesma, R. P.; Tomović, Ž. Catalysts for Isocyanate Cyclotrimerization. *ChemCatChem* **2023**, *15* (10). https://doi.org/10.1002/cctc.202201362.

(4) Lee, J. K.; Gould, G. L.; Rhine, W. Polyurea Based Aerogel for a High Performance Thermal Insulation Material. *J. Sol-Gel Sci. Technol.* **2009**, *49* (2), 209–220.

https://doi.org/10.1007/s10971-008-1861-6.

(5) Saeed, A. M.; Wisner, C. A.; Donthula, S.; Majedi Far, H.; Sotiriou-Leventis, C.; Leventis, N. Reuseable Monolithic Nanoporous Graphite-Supported Nanocatalysts (Fe, Au, Pt, Pd, Ni, and Rh) from Pyrolysis and Galvanic Transmetalation of Ferrocene-Based Polyamide Aerogels. *Chem. Mater.* **2016**, *28* (13), 4867–4877. https://doi.org/10.1021/acs.chemmater.6b02364.

(6) Leventis, N.; Chidambareswarapattar, C.; Bang, A.; Sotiriou-Leventis, C. Cocoon-in-Web-like Superhydrophobic Aerogels from Hydrophilic Polyurea and Use in Environmental Remediation. *ACS Appl. Mater. Interfaces* **2014**, *6* (9), 6872–6882. https://doi.org/10.1021/am500685k.

(7) Taghvaee, T.; Donthula, S.; Rewatkar, P. M.; Majedi Far, H.; Sotiriou-Leventis, C.; Leventis, N. K-Index: A Descriptor, Predictor, and Correlator of Complex Nanomorphology to Other Material Properties. *ACS Nano* **2019**, *13* (3), 3677–3690. https://doi.org/10.1021/acsnano.9b00396.

(8) Diascorn, N.; Calas, S.; Sallée, H.; Achard, P.; Rigacci, A. Polyurethane Aerogels Synthesis for Thermal Insulation – Textural, Thermal and Mechanical Properties. *J. Supercrit. Fluids* **2015**, *106*, 76–84. https://doi.org/10.1016/j.supflu.2015.05.012.

(9) Trifu, R.; Gould, G.; White, S. Flexible Polyisocyanate Based Aerogels. *MRS Adv.* **2017**, *325*, 1-. https://doi.org/10.1557/adv.201.

(10) Merillas, B.; Villafañe, F.; Rodríguez-Pérez, M. Á. Super-Insulating Transparent Polyisocyanurate-Polyurethane Aerogels: Analysis of Thermal Conductivity and Mechanical Properties. *Nanomaterials* **2022**, *12* (14). https://doi.org/10.3390/nano12142409.

(11) Tian, J.; Yang, Y.; Xue, T.; Chao, G.; Fan, W.; Liu, T. Highly Flexible and Compressible Polyimide/Silica Aerogels with Integrated Double Network for Thermal Insulation and Fire-Retardancy. *J. Mater. Sci. Technol.* **2022**, *105*, 194–202. https://doi.org/10.1016/j.jmst.2021.07.030.

(12) Ma, Z.; Xue, T.; Wali, Q.; Miao, Y. E.; Fan, W.; Liu, T. Direct Ink Writing of Polyimide/Bacterial Cellulose Composite Aerogel for Thermal Insulation. *Compos. Commun.* **2023**, *39* (January), 101528. https://doi.org/10.1016/j.coco.2023.101528.

(13) Tafreshi, O. A.; Ghaffari-Mosanenzadeh, S.; Karamikamkar, S.; Saadatnia, Z.; Kiddell, S.; Park, C. B.; Naguib, H. E. Novel, Flexible, and Transparent Thin Film Polyimide Aerogels with Enhanced Thermal Insulation and High Service Temperature. *J. Mater. Chem. C* **2022**, *10* (13), 5088–5108. https://doi.org/10.1039/d1tc06122d.

(14) Yao, K.; Jiang, S.; Li, S.; Zhang, C.; Hou, H. Solvothermal Imidization to Polyimide Composite Aerogels by Vacuum Drying. *Compos. Commun.* **2023**, *38* (December 2022), 101503. https://doi.org/10.1016/j.coco.2023.101503.

(15) Alshrah, M.; Tran, M. P.; Gong, P.; Naguib, H. E.; Park, C. B. Development of High-Porosity Resorcinol Formaldehyde Aerogels with Enhanced Mechanical Properties through Improved Particle Necking under CO2 Supercritical Conditions. *J. Colloid Interface Sci.* **2017**, *485*, 65–74. https://doi.org/10.1016/j.jcis.2016.09.030.

(16) Schwan, M.; Tannert, R.; Ratke, L. New Soft and Spongy Resorcinol-Formaldehyde Aerogels. *J. Supercrit. Fluids* **2016**, *107*, 201–208. https://doi.org/10.1016/j.supflu.2015.09.010.

(17) Ren, H.; Zhu, J.; Bi, Y.; Xu, Y.; Zhang, L. Facile Fabrication of Multifunctional Monolithic Polyamide Aerogels. *J. Porous Mater.* **2017**, *24* (5), 1165–1173. https://doi.org/10.1007/s10934-016-0356-z.

(18) Williams, J. C.; Meador, M. A. B.; McCorkle, L.; Mueller, C.; Wilmoth, N. Synthesis and Properties of Step-Growth Polyamide Aerogels Cross-Linked with Triacid Chlorides. *Chem. Mater.* **2014**, *26* (14), 4163–4171. https://doi.org/10.1021/cm5012313.
